# The complete mitochondrial genome of the blackskin catfish (*Clarias meladerma*: Clariidae) from Rokan River, Riau, Indonesia

**DOI:** 10.1080/23802359.2024.2392742

**Published:** 2024-08-19

**Authors:** Huria Marnis, Khairul Syahputra, Bambang Iswanto, Imam Civi Cartealy, Sularto, Jadmiko Darmawan, Erma Primanita Hayuningtyas, Rahmat Hidayat, Arsad Tirta Subangkit, Arianto

**Affiliations:** a Research Center for Fishery, National Research and Innovation Agency (BRIN), Cibinong, Indonesia; b Research Center for Computation, National Research and Innovation Agency (BRIN), Cibinong, Indonesia

**Keywords:** High-throughput sequencing, Indonesian catfish, mitogenome, phylogenetic position, siluriformes

## Abstract

*Clarias meladerma* Bleeker, 1846, a native catfish species in Indonesia belonging to the family Clariidae. The present study the complete mitochondrial genome sequence of *C. meladerma* from the Rokan River was sequenced by using next-generation sequencing, and its phylogenetic relationship was analyzed. The mitochondrial genome comprises 13 protein-coding genes (PCGs), 22 tRNA genes, and two rRNA genes, with a total length of 16,808 bp. The mitogenome of *C. meladerma* exhibits a base composition of 32.49% adenine, 25.75% thymine, 14.51% guanine, and 27.25% cytosine. Phylogenetic analysis indicated that *C. meladerma* has the same clade with *C. macrocephalus, C. batrachus*, and *C. fucus*. In essence, the findings of this study lay down a genetic foundation for future investigations into *C. meladerma*.

## Introduction

The catfish, *Clarias meladerma* Bleeker, 1846, a member of the Clariidae family and commonly known as the blackskin catfish, holds economic significance in Indonesia. This species is found in lowland wetlands, bogs, and peats across East Sumatra and South Kalimantan (Kottelat et al. 1993). The Clarias genus comprises 62 taxonomically accepted species (Froese and Pauly 2023), with only 8 of them having complete mitochondrial DNA sequences deposited in the GenBank database. Recently, *C. meladerma* has been categorized as ‘least concern’ with decreasing population trends due to the ongoing decline in the area, extent, and/or quality of its habitat, as per the International Union for the Conservation of Nature (IUCN 2021). Given its economic importance and the limited genetic research conducted, there is a need to study this species further, including evaluating its phylogenetic placement within the Clariidae family. Presently, research on this species is primarily limited to morphological characterization, emphasizing the importance of molecular studies for a comprehensive understanding.

Efforts to deduce the phylogenetic relationships within Clariidae fish through molecular approaches have been undertaken (Kushwaha et al. 2015; Zhou et al. 2015; Yang et al. 2016). However, these analyses did not include *C. meladerma*. Consequently, the phylogenetic connections of this species with other Clarias species remain unknown. The primary aim of this study was to acquire the complete mitochondrial genome of *C. meladerma* using high-throughput sequencing technology and assess its phylogenetic relationships among other species within the Clarias genus. This investigation marks the first genomic exploration of the species. The outcomes of this study will also make substantial contributions to genomic references for theClariidae family.

## Materials and methods

**Figure 1.**
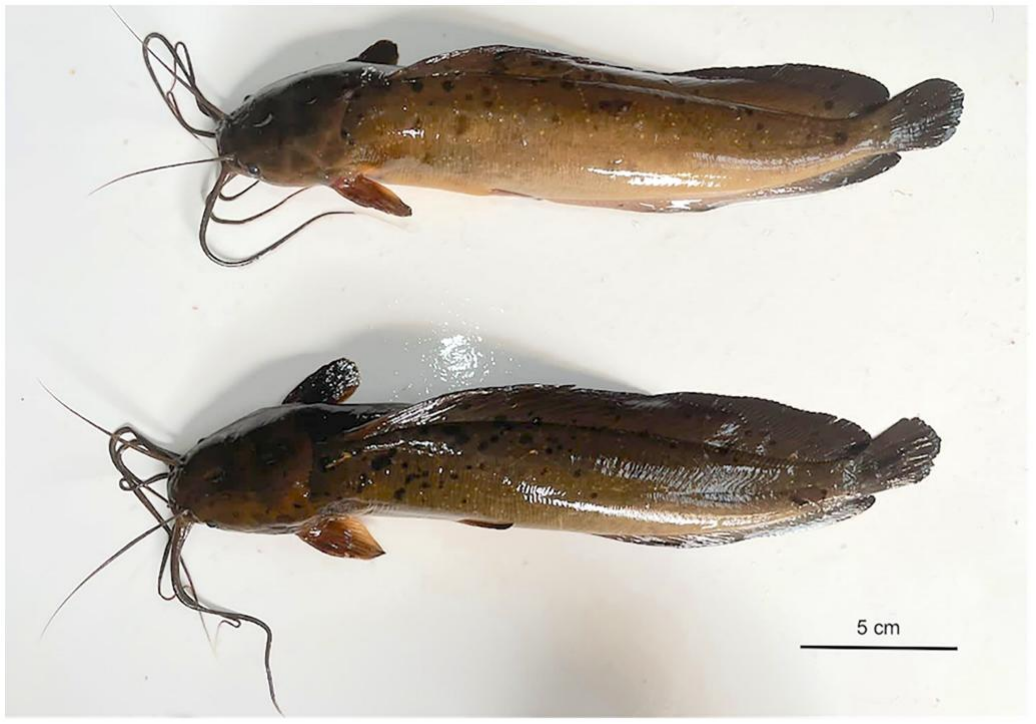
Photograph of *Clarias meladerma*, female (top), male (bottom). Photo: Huria Marnis and Khairul Syahputra.

Live specimens of *C. meladerma* (Figure 1) were procured from the Rokan River near Ujung Batu district, Riau Province, Indonesia. The collection took place on December 31, 2023 utilizing a cast net. Geographic coordinate position of the sampling site was 0°42’13.9” N, 100°30’51.3” E. Female fish was utilized for this study. The morphological characterization of the sample fish was conducted using morphometric and meristic characters, following standard methods for identifying *Clarias meladerma* (Sudarto and Pouyaud 2005; Sudarto 2007). The fish sample showed that the body was compressed, featuring a large head, where the head length was 21.3% and head width was 16.3% of standard length. The number of gill rakers on the first branchial arch ranged from 20. The distance between the tip of the occipital process and the base of the first dorsal fin ray was remarkably short, measuring 2.2% of the standard length. The anterior margin of the pectoral spine was strongly serrated, with 15 serrations. Additionally, black blotches were present on the head, dorsal and anal fins, and lateral flanks of the body. The specimen underwent formal submission to the Directorate of Scientific Collection Management at Gedung B.J. Habibie Jalan M.H. Thamrin Nomor 8, Jakarta Pusat 10340, Research and Innovation National Agency (BRIN) (https://www.brin.go.id, Darmawan, dit-pki@brin.go.id) under voucher number MBZ.FISH.26926.

The genomic DNA was extracted from fin clip samples using the DNAeasy Blood and Tissue Kit (QIAGEN, Hilden, Germany) following the manufacturer’s instructions and stored at −20 °C. The existence of intact DNA molecules, with lengths exceeding 10,000 base pairs and the total concentration of genomic DNA obtained, greater than 30 ng/ul, was validated through gel electrophoresis utilizing a 1.5% agarose gel stained with ethidium bromide. The quantification of DNA was performed using a NanoDrop 2000 Spectrophotometer (Thermo Fisher Scientific, Waltham, MA, USA). To ensure accurate DNA concentration measurement, QubitTM dsDNA HS Assay Kit and the QubitVR 2.0 fluorometer (Life Technologies, United States). High-quality genomic DNA was utilized to construct a DNA library.

The sequencing process was conducted at the Genomic Laboratory, National Research and Innovation Agency, Cibinong, Indonesia utilizing the ELSA-BRIN Point mechanism. The long reads were generated through PromethION sequencing (Nanopore, Oxford, UK). The sequencing library was prepared using the ligation sequencing kit (SQK-LSK110). Each library was run in an FLO-PRO002 flow cell, according to the manufacturer’s instructions, for 48 h. Default parameters were used for all software unless otherwise specified. The run was base called live with default settings (MinKNOW Core version 5.4.3, Bream version 7.4.8, and Guppy version 6.4.6) (Oxford Nanapore Technologies). Reads <4000 bp and quality scores of <7 were discarded for downstream analysis, 18.23 Gb of sequence data were generated from PromethION sequencing (Nanopore, Oxford, UK). Raw nanopore reads underwent assembly by using Flye v2.9.2 (Kolmogorov et al. 2019). Mitochondrial genome was then extracted from assembled reads by using GetOrganelle v1.7.7.0 (Jian et al. 2020). To enhance the accuracy of assembled mitochondrial genome, we performed two rounds of polishing using NextPolish (Hu et al. 2019). The BAM file was generated by mapping the reads to the final assembly using Minimap2 v2.24 (Li 2018). Then, a coverage mitogenome plot was generated using bam2plot (Ros 2023). The read coverage depth map is shown in Supplementary Figure S1. Mitochondrial genome annotation and visualization was performed in MitoAnnotator v 3.99 (Iwasaki et al. 2013; Zhu et al. 2023). Protein coding gene start and stop codons were verified in Geneious Prime 2020.1(Kearse et al. 2012).

The complete mitochondrial genome of *C. meladerma* was compared against the GenBank database in NCBI, and mitogenomes from seven species highly similar to *C. meladerma*, with a Max score ranging between 18,757 and 17,439 with percent identity (90.95%-89.16%), were selected to phylogenetic analysis. The following sequences were used were utilized including *C. macrocephalus* (Duong et al. 2020), *C. fuscus* (Yang et al. 2016), *C. batrachus* (Kushwaha et al. 2015), *Clarias* sp (Nakatani et al. 2011), *C. gariepinus* (Han et al. 2015), *C. dussumieri* (unpublished), and *C. camerunensis* (De Alwis et al. 2023). Two catfish species from different families, namely *Heteropneustes fossilis* (Sahoo et al. 2016) and *Pangasius nasutus* (unpublished), which are more distantly related to the target species in the present study were used as outgroups. The complete mitogenome sequence of *C. meladerma* together with above nine comparison species was aligned using clustalW in MEGA11 with default settings, and then followed by a phylogenetic analysis using the Maximum likelihood (ML) method with 1000 bootstrap replicates (Tamura et al. 2021).

## Results

**Figure 2.**
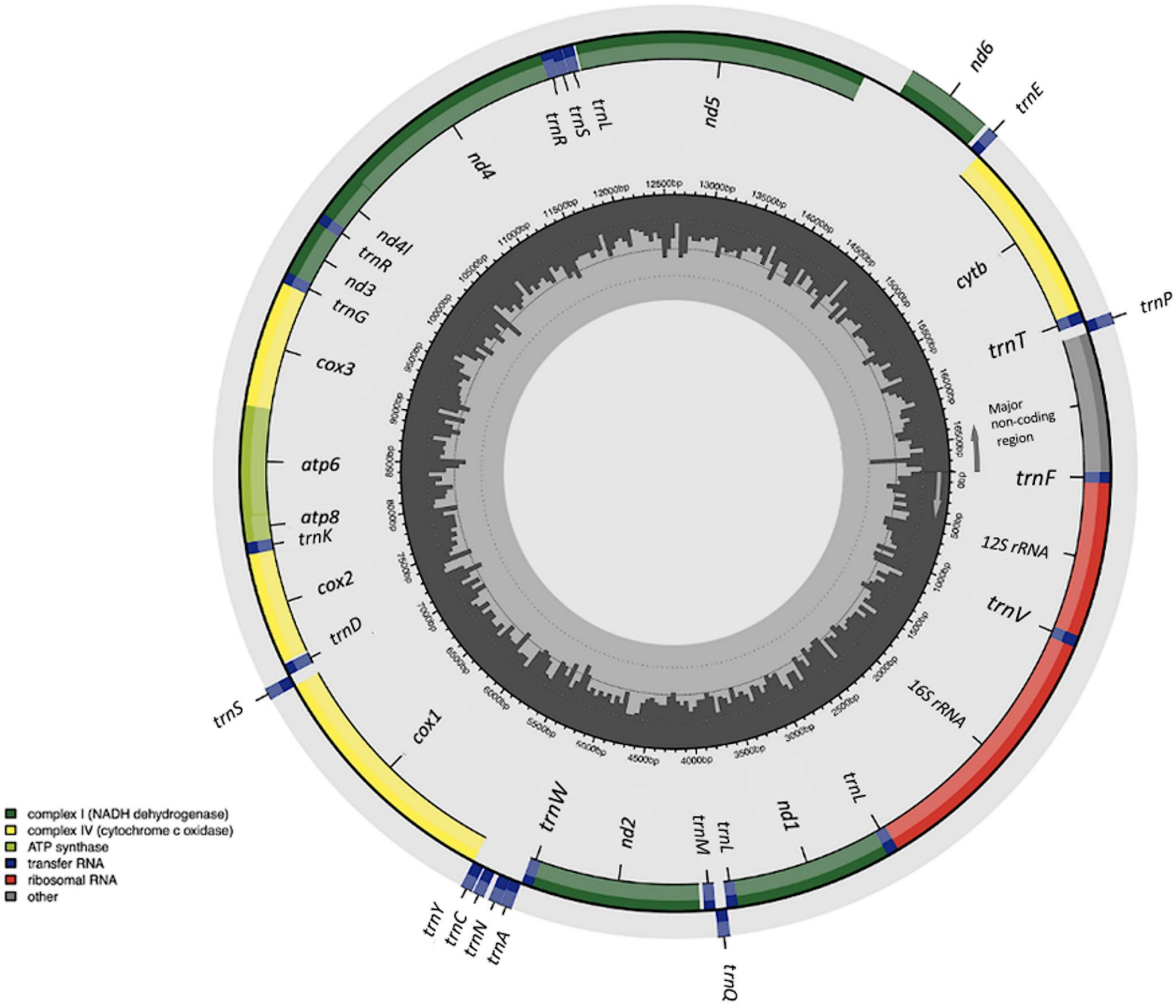
The circular mitochondrial genome map of *Clarias meladerma*. Genes oriented in the reverse direction are depicted in the outermost concentric ring, while those in the forward orientation are situated in the second outermost ring. The innermost rings of the image illustrate the percentage of GC content for every 5 base pairs of the mitogenome, with longer lines indicating higher GC percentages.

The mitogenome of *C. meladerma* (accession number: PP109374) from the Rokan River population, as presented in Figure 2, is 16,808 bp in length, containing 13 PCGs genes, 22 tRNA genes, 2 rRNA genes, and 862 bp major non-coding region between *trnP* and *trnF*. The base composition of the *C. meladerma* mitogenome is 32.49% A, 25.75% T, 14.51% G, and 27.25% C. The 13 protein-coding genes within the mitochondria have a common initiation codon, ATG. The nucleotide sequence 'TAA', which serves as a complete stop codon, is observed in the genes *nd1*, *cox1*, *atp8*, and *nd4l*. Additionally, an incomplete stop codon is identified in the genes *cox2*, *cox*3, *atp6*, *nd4*, and *cytb*. Among the protein-coding genes, the *nd5* gene is the longest at 1,827 base pairs (bp), while the *atp8* gene is the shortest at 168 bp. There is additional non-coding region between *nd5* and *nd6* spanning 239 bp in length (Figure 2).

**Figure 3.**
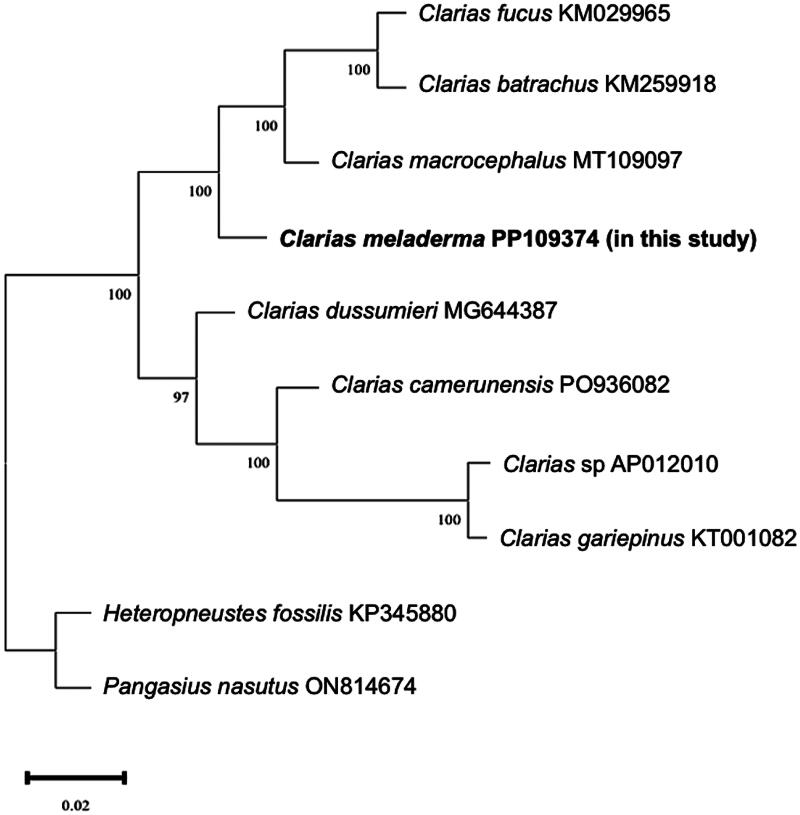
Phylogenetic tree of mitogenome *C. meladerma* and seven other clarias mitogenome of species produced from maximum likelihood. Two species of *Heteropneustes fossilis* and *Pangasius nasutus* is designated to be outgroups. The numbers on the branches are bootstrap percentages based on 1000 replicates. The scientific name and its corresponding GenBank accession number are provided for each individual species. The following sequences is used *C. fuscus* acc *C.* KM029965 (Yang et al. 2016), *C. batrachus* acc KM259918 (Kushwaha et al. 2015), *Macrocephalus* acc MT109097 (Duong et al. 2020), *C. meladerma* acc PP109374 (this study), *C. dussumieri* acc MG644387 (unpublished), *C. camerunensis* acc OP936082 (De Alwis et al. 2023), *Clarias* sp acc AP012010 (Nakatani et al. 2011), *C. gariepinus* acc KT001082 (Han et al. 2015), *Heteropneustes fossilis* acc KP345880 (Sahoo et al. 2016), and *Pangasius nasutus* acc ON814674 (unpublished).

The phylogenetic analysis illustrates the relationship of mitogenome among eight species of Clarias and out group species. The maximum likelihood tree depicted in Figure 3 demonstrates a clear division of Clarias species into two separate clades, in which *C. meladerma*, *C. macrocephalus*, *C. batrachus*, and *C. fucus* are grouped in one clade.

## Discussion and conclusions

We successfully sequenced and assembled the complete mitogenome of *C. meladerma* for the first time using high-throughput sequencing (Oxford Nanapore Technologies). The mitogenome of *C. meladerma* exhibited the characteristic arrangement observed in other vertebrates (Dysin et al. 2022). The *C. meladerma* mitogenome was distinct from other Clariidae mitogenomes analyzed in the present study. The pylogenetic result of this study was in line with previous research showing that *C. fuscus*, *C. batrachus*, *C. macrocephalus*, and *C. meladerma* were closely related, using genomic DNA analysis (cross-amplification of microsatellite loci) (Nazia and Siti Azizah 2014). Our findings provide a valuable reference for conservation efforts focused on *C. meladerma* populations from the Rokan River and offer a deeper understanding of the mitochondrial genetic relationships among Clarias species.

## Supplementary Material

Supplementary Figure S1.tiff

Supplementary material.pdf

Supplementary material.docx

## Data Availability

The genome sequence data that support the findings of this study are openly available in GenBank of NCBI at (https://www.ncbi.nlm.nih.gov/) under accession number PP109374). The associated BioProject, Bio-Sample and SRA numbers were PRJNA1127784, SAMN42019558, and SRR29734923, respectively.

## References

[CIT0001] De Alwis PS, Kundu S, Gietbong FZ, Amin MHFa, Lee S-R, Kim H-W, Kim AR. 2023. Mitochondriomics of Clarias sishes (Siluriformes: Clariidae) with a new assembly of Clarias camerunensis: insights into the genetic characterization and diversification. Life. 13(2):482. https://www.mdpi.com/2075-1729/13/2/482. doi:10.3390/life13020482. 36836839 PMC9960581

[CIT0002] Duong T-Y, Tan MH, Lee YP, Croft L, Austin CM. 2020. Dataset for genome sequencing and de novo assembly of the Vietnamese bighead catfish (Clarias macrocephalus Günther, 1864). Data Brief. 31:105861. doi:10.1016/j.dib.2020.105861. 32637481 PMC7326715

[CIT0003] Dysin AP, Shcherbakov YS, Nikolaeva OA, Terletskii VP, Tyshchenko VI, Dementieva NV. 2022. Salmonidae genome: features, evolutionary and phylogenetic characteristics. Genes (Basel). 13(12):2221. doi:10.3390/genes13122221. 36553488 PMC9778375

[CIT0004] Froese R, Pauly D. 2023. *FishBase*. www.fishbase.org.

[CIT0005] Han C, Li Q, Xu J, Li X, Huang J. 2015. Characterization of Clarias gariepinus mitochondrial genome sequence and a comparative analysis with other catfishes. Biologia. 70(9):1245–1253. doi:10.1515/biolog-2015-0145.

[CIT0006] Hu J, Fan J, Sun Z, Liu S. 2019. NextPolish: a fast and efficient genome polishing tool for long-read assembly. doi:10.1093/bioinformatics/btz891. 31778144

[CIT0007] IUCN. 2021. The IUCN red list of threatened species. Version 2021‐1. IUCN Red List of Threatened Species [accessed 2022 Dec 8]. www.iucnredlist.org/.

[CIT0008] Iwasaki W, Fukunaga T, Isagozawa R, Yamada K, Maeda Y, Satoh TP, Sado T, Mabuchi K, Takeshima H, Miya M, et al. 2013. MitoFish and MitoAnnotator: a mitochondrial genome database of fish with an accurate and automatic annotation pipeline. Mol Biol Evol. 30(11):2531–2540. doi:10.1093/molbev/mst141. 23955518 PMC3808866

[CIT0009] Jian J-J, Yu W-B, Yang J-B, Song Y, Yi T-S, Li D-Z. 2020. GetOrganelle: a fast and versatile toolkit for accurate de novo assembly of organelle genomes. Genome Biol. 21(1):1–31. doi:10.1186/s13059-020-02154-5 PMC7488116 32912315

[CIT0010] Kearse M, Moir R, Wilson A, Stones-Havas S, Cheung M, Sturrock S, Buxton S, Cooper A, Markowitz S, Duran C, et al. 2012. Geneious basic: an integrated and extendable desktop software platform for the organization and analysis of sequence data. Bioinformatics. 28(12):1647–1649. doi:10.1093/bioinformatics/bts199. 22543367 PMC3371832

[CIT0011] Kolmogorov M, Yuan J, Lin Y, Pevzner PA. 2019. Assembly of long, error-prone reads using repeat graphs. Nat Biotechnol. 37(5):540–546. doi:10.1038/s41587-019-0072-8. 30936562

[CIT0012] Kottelat M, Whitten A, Kartikasari S, Wirjoatmodjo S. 1993. Freshwater fishes of Western Indonesia and Sulawesi. Hong Kong: Periplus Editions; p. 221.

[CIT0013] Kushwaha B, Kumar R, Agarwal S, Pandey M, Nagpure NS, Singh M, Srivastava S, Joshi CG, Das P, Sahoo L, et al. 2015. Assembly and variation analyses of *Clarias batrachus* mitogenome retrieved from WGS data and its phylogenetic relationship with other catfishes. Meta Gene. 5:105–114. doi:10.1016/j.mgene.2015.06.004. 26137446 PMC4484717

[CIT0014] Li H. 2018. Minimap2: pairwise alignment for nucleotide sequences. Bioinformatics. 34(18):3094–3100. doi:10.1093/bioinformatics/bty191. 29750242 PMC6137996

[CIT0015] Nakatani M, Miya M, Mabuchi K, Saitoh K, Nishida M. 2011. Evolutionary history of Otophysi (Teleostei), a major clade of the modern freshwater fishes: pangaean origin and Mesozoic radiation. BMC Evol Biol. 11(1):177. doi:10.1186/1471-2148-11-177. 21693066 PMC3141434

[CIT0016] Nazia A, Siti Azizah M. 2014. Isolation of microsatellites in the bighead catfish, Clarias macrocephalus and cross-amplification in selected Clarias species. Mol Biol Rep. 41(3):1207–1213. doi:10.1007/s11033-013-2965-9. 24381108

[CIT0017] Ros W. 2023. bam2plot. https://github.com/willros/bam2plot.

[CIT0018] Sahoo L, Kumar S, Das SP, Patnaik S, Bit A, Sundaray JK, Jayasankar P, Das P. 2016. Complete mitochondrial genome sequence of Heteropneustes fossilis obtained by paired end next generation sequencing. Mitochondrial DNA Part A. 27(4):2485–2486. doi:10.3109/19401736.2015.1033710. 26016883

[CIT0019] Sudarto H. 2007. Systematic revision and phylogenetic relationships among populations of Clariid species in Southeast Asia [Thesis]. University of Indonesia, Depok, West Java, Indonesia.

[CIT0020] Sudarto S, Pouyaud L. 2005. Identification key based on morphological characters of the southeast Asian species of the genus Clarias (Pisces: Clarhdae). Jurnal Iktiologi Indonesia. 5(2):39–47.

[CIT0021] Tamura K, Stecher G, Kumar S. 2021. MEGA11: molecular evolutionary genetics analysis version 11. Mol Biol Evol. 38(7):3022–3027. doi:10.1093/molbev/msab120. 33892491 PMC8233496

[CIT0022] Yang H, Sun J, Zhao H, Chen Y, Yang Z, Li G, Liu L. 2016. The complete mitochondrial genome of the Clarias fuscus (Siluriformes, Clariidae). Mitochondrial DNA Part A. 27(2):1255–1256. doi:10.3109/19401736.2014.945544. 25090377

[CIT0023] Zhou C, Wang X, Guan L, He S. 2015. The complete mitochondrial genome of Clarias fuscus (Teleostei, Siluriformes: Clariidae). Mitochondrial DNA. 26(2):270–271. doi:10.3109/19401736.2013.823177. 24460162

[CIT0024] Zhu T, Sato Y, Sado T, Miya M, Iwasaki W. 2023. MitoFish, MitoAnnotator, and MiFish pipeline: updates in 10 years. Mol Biol Evol. 40(3):msad035. doi:10.1093/molbev/msad035. PMC9989731 36857197

