# Supplementary material for: The complete mitochondrial genome of the blackskin catfish (Clarias meladerma: Clariidae) from Rokan River, Riau, Indonesia
Source: Mitochondrial DNA B Resour. 2024 Aug 19;9(8):1093–7. doi: 10.1080/23802359.2024.2392742 (PMC11334743; doi:10.1080/23802359.2024.2392742)
Supplement: Supplementary material.pdf [file TMDN_A_2392742_SM1776.pdf]

Supplementary Material

The complete mitochondrial genome of the blackskin catfish (*Clarias meladerma*: Clariidae) from Rokan River, Riau, Indonesia

Huria Marnis[a], Khairul Syahputra[a], Bambang Iswanto[a], Imam Civi Cartealy[b], Sularto[a], Jadmiko Darmawan[a], Erma Primanita Hayuningtyas[a], Rahmat Hidayat[a], Arsad Tirta Subangkit[a], Arianto[a]

[a]Research Center for Fishery, National Research and Innovation Agency (BRIN), Cibinong, Indonesia, 16911; [b]Research Center for Computation, National Research and Innovation Agency (BRIN), Cibinong, Indonesia, 16911

Corresponding author: Huria Marnis (marnis.huria@gmail.com/huria.marnis@brin.go.id), Research Center for Fishery, National Research and Innovation Agency (BRIN), Cibinong, Indonesia, 16911

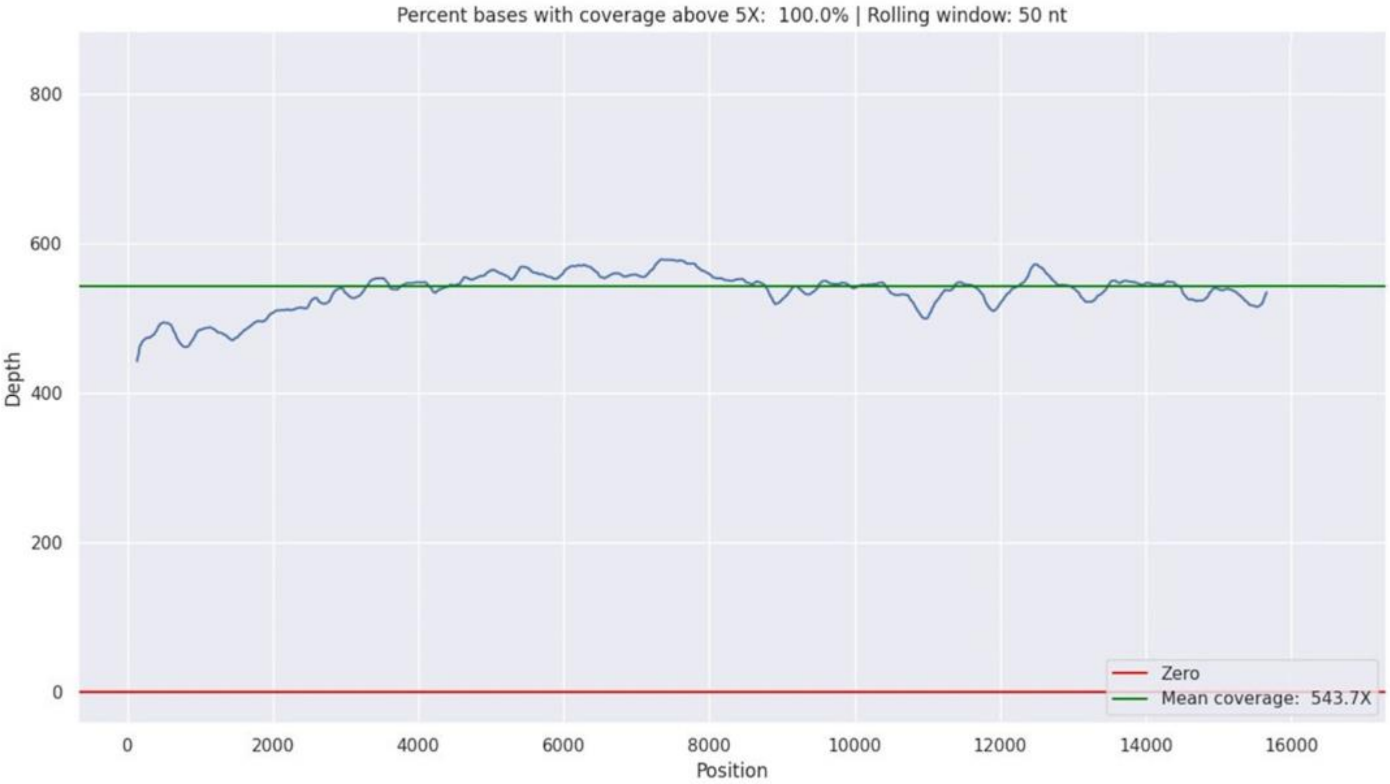

Supplementary Figure S1 - Read coverage depth map of blackskin catfish (*Clarias meladerma*: Clariidae) mitogenome from Rokan River, Riau, Indonesia.
