# Supplementary material for: The complete mitochondrial genome of the blackskin catfish (Clarias meladerma: Clariidae) from Rokan River, Riau, Indonesia
Source: Mitochondrial DNA B Resour. 2024 Aug 19;9(8):1093–7. doi: 10.1080/23802359.2024.2392742 (PMC11334743; doi:10.1080/23802359.2024.2392742)
Supplement: Supplementary material.docx [file TMDN_A_2392742_SM1775.docx]

**Supplementary Material**

**The complete mitochondrial genome of the blackskin catfish (*Clarias meladerma*: Clariidae) from Rokan River, Riau, Indonesia**

Huria Marnis^a^, Khairul Syahputra^a^, Bambang Iswanto^a^, Imam Civi Cartealy^b^, Sularto^a^, Jadmiko Darmawan^a^, Erma Primanita Hayuningtyas^a^, Rahmat Hidayat^a^, Arsad Tirta Subangkit^a^, Arianto^a^

^a^Research Center for Fishery, National Research and Innovation Agency (BRIN), Cibinong, Indonesia, 16911; ^b^Research Center for Computation, National Research and Innovation Agency (BRIN), Cibinong, Indonesia, 16911

Corresponding author: Huria Marnis (marnis.huria@gmail.com/huria.marnis@brin.go.id), Research Center for Fishery, National Research and Innovation Agency (BRIN), Cibinong, Indonesia, 16911

*
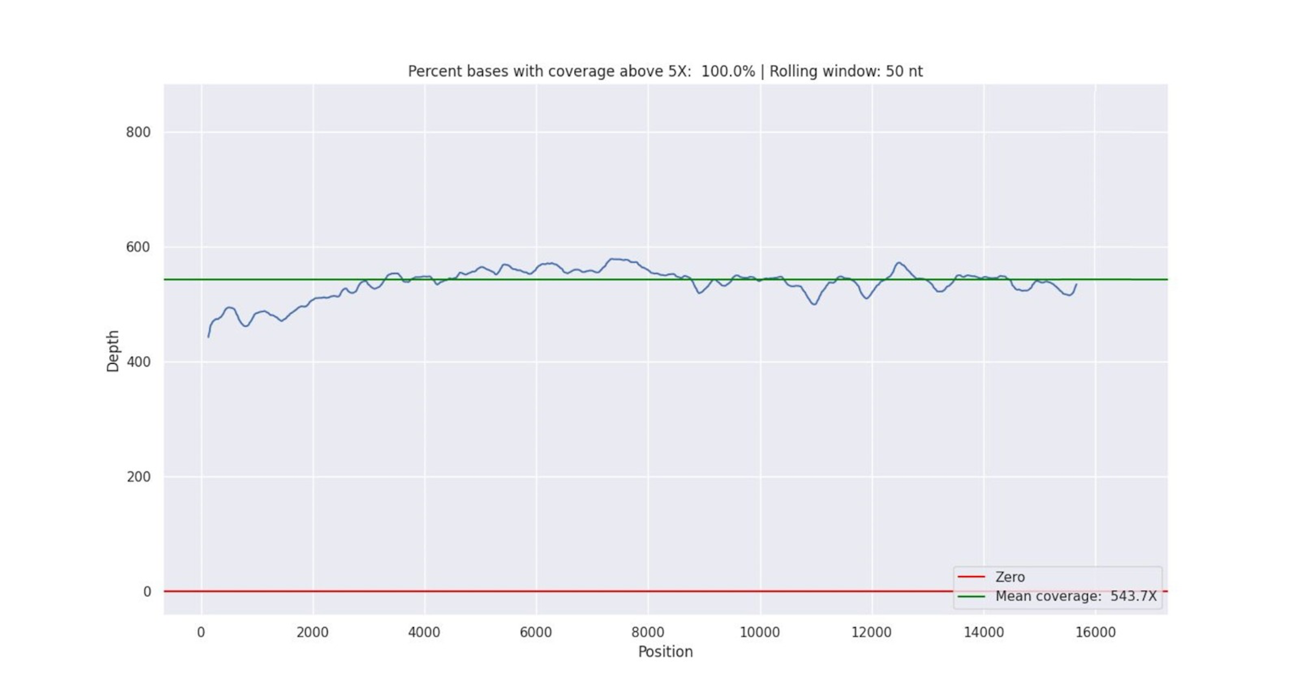
*

Supplementary Figure S1 - Read coverage depth map of blackskin catfish (*Clarias meladerma*: Clariidae) mitogenome from Rokan River, Riau, Indonesia.
